# Association of Visual Impairment with Psychological Distress in Older Adults: A Survey of 105,092 Older People in Taiwan

**DOI:** 10.3390/jcm11051458

**Published:** 2022-03-07

**Authors:** Chu-Yu Yen, I-Mo Fang, Hsiao-Yun Hu, Shih-Han Weng

**Affiliations:** 1 Department of Ophthalmology, Taipei City Hospital, Ren-Ai Branch, Taipei 10629, Taiwan; dax65@tpech.gov.tw; 2 Department of Ophthalmology, Taipei City Hospital, Zhongxiao Branch, Taipei 10002, Taiwan; 3 Department of Ophthalmology, National Taiwan University Hospital, Taipei 11556, Taiwan; 4 Department of Special Education, University of Taipei, Taipei 11153, Taiwan; 5 Department of Education and Research, Taipei City Hospital, Taipei 10629, Taiwan; a3547@tpech.gov.tw (H.-Y.H.); a4391@tpech.gov.tw (S.-H.W.); 6 Institute of Public Health, National Yang Ming Chiao Tung University, Taipei 11221, Taiwan

**Keywords:** visual impairment, psychological distress, living status, marital status, educational status, smoking habits, body mass index

## Abstract

This study aimed to evaluate the association between visual impairment (VI) and psychological distress (PD) among older adults in Taiwan. The present cohort study included participants aged >65 years who participated in a physical examination program. Participants were divided into two groups on the basis of whether they had PD at baseline. The association between PD and VI with other variables was compared using the two-sample *t*-test for continuous variables and chi-squared test for discrete variables. Cox regression analyses were used to calculate the hazard ratio (HR). Cumulative incidence of PD was analyzed using the Kaplan-Meier method, and differences among participants with different severities of VI were analyzed using the two-tailed log-rank test. Subgroup analyses were performed to calculate the HR for PD among participants with different severities of VI. The PD group showed a significantly high percentage of VI. In addition, participants with VI showed a significantly higher HR and seven-year cumulative incidence rate of PD than those without VI. VI was independently and significantly associated with a higher incidence of PD among older Asian people. Therefore, identifying and treating correctible VI is important to prevent PD and improve the overall quality of life.

## 1. Introduction

Psychological distress (PD) is a common mental health problem in modern society. The prevalence rates of depressive symptoms and anxiety disorders of the global population are 4.4% and 3.6%, respectively [1]. Of the top 20 causes of the global burden of disease in 2013, 5 types of PD, including major depression, anxiety disorders, schizophrenia, dysthymia, and bipolar disorder, have been reported [2]. PD is not only a mental health problem but is also associated with a deleterious effect on physical health and the quality of life (QOL). In a study conducted in the United Kingdom, PD increased the risk of all-cause mortality by 21%, cardiovascular disease death by 22%, cancer death by 9%, and death due to external causes by 26% [3]. Another population survey conducted in Singapore reported that PD is associated with heart disease, diabetes, arthritis, renal failure, and lung disease [4]. Given the significant influence of PD on general health care, identifying and managing modifiable risk factors related to this issue are important to improve the QOL and relieve social burdens.

Visual impairment (VI), including low vision and blindness, affects a substantial proportion of the older population worldwide. In 2017, more than 7 million Americans had VI, of which 1 million were blind. In addition, 93 million US adults are at risk for severe vision loss [5]. A study conducted in Taiwan has revealed that the prevalence of VI in older patients aged >55 years is high at 11.0% [6]. This prevalence rate may increase because of the aging population and the increased incidence of chronic diseases. Most VIs are due to refractive errors and cataracts, which could be corrected with prescription glasses and cataract surgery, respectively [7,8]. However, most people view VI as a natural result of advancing age; thus, they do not seek appropriate medical care, although these impairments are mostly treatable [9]. In PD, VI is also associated with higher mortality. The risk of mortality is 29% higher for patients with mild vision impairment compared with those with normal vision. The risk increases to 89% among those with severe vision impairment [10]. Possible etiologies include increased risk of falls, loss of independence, depression, disability, cognitive impairment, and dementia [11,12].

The association between VI and PD has been widely investigated. In a study conducted by the Centers for Disease Control and Prevention, one in four adults with VI had PD. People with VI have a higher risk of serious PD, particularly those who are younger, nonsmokers, and physically inactive and have arthritis or chronic obstructive pulmonary disease [13]. The prevalence of depression and anxiety is significantly higher in older adults with VI, with agoraphobia and social phobia representing the most prevalent anxiety disorders [14,15]. The possible etiologies of this condition may include greater activity limitations, functional capacity loss, financial strain, reduced participation in pleasurable activities, low self-efficacy, and reduced social integration [16,17,18,19]. Despite the correlations between VI and PD, few studies have comprehensively discussed the different severities of VI, the influence of social status, baseline lab data, and their association with PD. The purpose of this study is two-fold. First, we aimed to evaluate the association among different severities of VI and PD. Second, we sought to evaluate the modifiable risk factors of PD on the basis of social status and systemic personal health conditions obtained from a physical examination database in Taiwan. The results of this study will highlight the importance of identifying and treating patients with VI to improve the overall QOL through PD control.

## 2. Participants and Methods

### 2.1. Study Population

**Figure 1:**
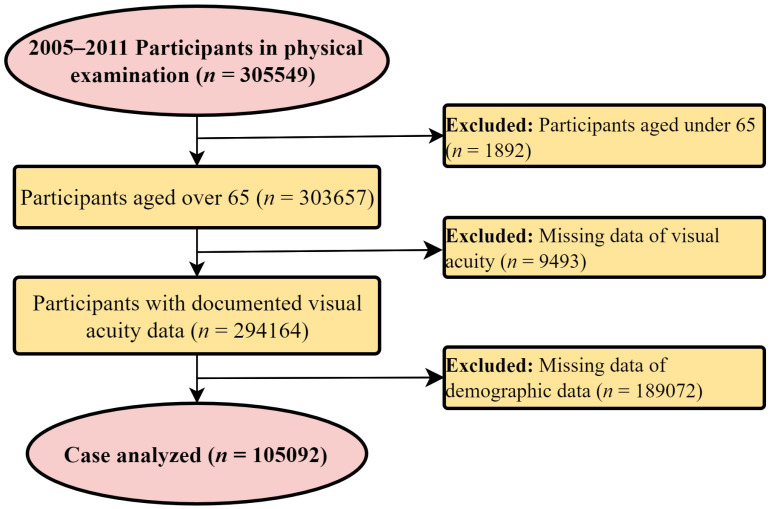
Flowchart of the participants included in the study.

The Taiwanese government has been implementing a standard annual physical examination program for the older adult population in Taipei City since 1973. Initially, Taipei citizens aged >65 years were eligible to voluntarily participate annually at no cost. However, the age-related eligibility criterion has been revised to ≥55 years for Taiwanese aborigines. The present study included all participants of this program from 2005 to 2012 (*n* = 305,549). We excluded citizens aged <65 years (*n* = 1892) and those with missing visual acuity (VA; *n* = 9493) and demographic (*n* = 189,072) data. Thus, data from a total of 105,092 cases were included in our analyses (Figure 1). Data regarding participant identification were removed to ensure participant anonymity throughout the study period. The study adhered to the tenets of the Declaration of Helsinki, and it was approved by the Institutional Review Board of Taipei City Hospital (IRB No.: TCHIRB-10703110-W). Furthermore, written informed consent was obtained from all participants.

### 2.2. Materials and Variable Definitions

A longitudinal population-based cohort study was conducted to evaluate the risk and prevalence rates of VI and PD in older adults. VI was evaluated by measuring the distance presenting VA in the better-seeing eye under normal luminance with a Snellen chart at a distance of 6 m (20 feet). The definitions of VI and blindness were categorized on the basis of the World Health Organization (WHO) guidelines: mild VI (VA, 6/12–6/18), moderate VI (VA, 6/18–6/60), severe VI (VA, 6/60–3/60), and blindness (VA, <3/60). Moderate and severe VIs were defined as low vision [20]. For PD evaluation, the Brief Symptom Rating Scale-5 (BSRS-5) was used. The BSRS-5 is a 5-item, self-administered questionnaire in Chinese that is derived from the 50-item Brief Symptom Rating Scale, which measures anxiety (feeling tense or high-strung), depression (feeling depressed or in a low mood), hostility (feeling easily annoyed or irritated), interpersonal sensitivity (feeling inferior to others), and additional symptoms (having trouble falling asleep in the past week). The score for each item ranges from 0 to 4 (0, not at all; 1, a little bit; 2, moderately; 3, quite a bit; and 4, extremely). Patients with BSRS-5 scores of ≥6 were considered to have PD. The internal consistency coefficient for BSRS-5 was 0.84, and the area under the receiver-operating characteristic curve was 0.91. The sensitivity and specificity for PD were 82.6% and 81.8%, respectively [21,22]. We collected baseline VA and BSRS-5 scores for each participant. Demographic data, including sex, age, body mass index (BMI), living status, marital status, educational status, diabetes mellitus (DM), hypertension, blood cholesterol/triglyceride (TG)/albumin/globulin level, and smoking/alcohol consumption/betel nut chewing in the past six months, were also collected. DM was defined as fasting blood sugar level ≥126 mg/dL, self-report of physician-diagnosed DM, or the use of hypoglycemic medications. Hypertension was defined as blood pressure >140/90 mm Hg, self-report of physician-diagnosed hypertension, or the use of antihypertension medications. Abnormal blood cholesterol level was defined as serum total cholesterol level ≥200 mg/dL. Abnormal blood TG level was defined as serum TG level ≥200 mg/dL. Abnormal blood albumin level was defined as serum albumin <3.5 g/dL or self-report of physician-diagnosed hypoalbuminemia. Abnormal blood globulin level was defined as serum globulin level >3.5 g/dL or self-reported physician-diagnosed hyperglobulinemia.

### 2.3. Statistical Analysis

Participants were divided into two groups on the basis of whether they had PD at baseline. The association between PD and VI with other variables was compared using the two-sample *t*-test for continuous variables and chi-squared test for discrete variables. For patients who were PD-naive at baseline, univariate and multivariate Cox regression analyses were used for hazard ratio (HR) calculations to adjust for the baseline confounding factors for PD. Cumulative incidence of PD was analyzed using the Kaplan–Meier method, and differences among participants with normal vision, low vision, and blindness were analyzed using the two-tailed log-rank test. The time of entry to the program was the initial examination date (i.e., between 2005 and 2012), and the time of exit to the program was the end of follow-up (31 December 2012) or the date of PD diagnosis, if earlier. After stratifying according to sex; age; BMI; living status; marital status; educational status; hypertension; DM; and cholesterol, triglyceride, albumin, and globulin levels, subgroup analyses were performed to calculate HRs for PD among participants with low vision, and the results were compared with those of older people with normal vision. In addition, blindness was compared with those with normal vision. All *p*-values were two-sided, and *p*-values of <0.05 were considered statistically significant. SAS for Windows 9.3 (SAS Institute, Inc., Cary, NC, USA) was used for all analyses.

## 3. Results

**Figure 2:**
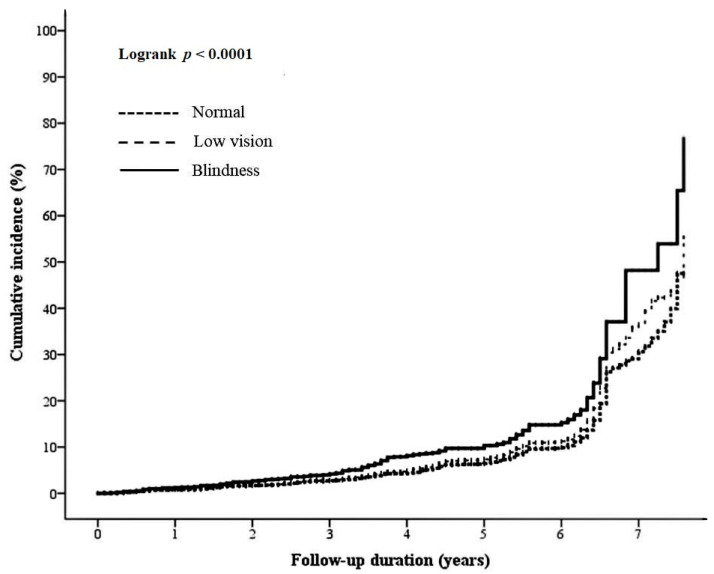
Seven-year cumulative incidence rates of PD, as demonstrated by PD development among groups with normal vision, low vision, and blindness (*p* < 0.0001). PD = psychological distress.

Participant demographics categorized on the basis of the presence of PD are listed in Table 1. Among the participants, 10,708 (10.19%) had PD, whereas 94,384 (89.81%) did not. Among the participants with PD, 22.92% had low vision, whereas 1.75% had blindness. By contrast, among the participants without PD, 19.52% had low vision, whereas 1.21% had blindness. The PD group showed significantly higher percentages of participants with low vision and blindness than the non-PD group (*p* < 0.0001, chi-squared test). Univariate Cox regression analysis revealed that participants with VI had higher HRs for PD (low vision, HR: 1.16, 95% confidence interval [CI]: 1.11–1.22; blindness, HR: 1.59, 95% CI: 1.37–1.83; *p* < 0.0001; Table 2). After adjusting for the covariates during multivariate Cox regression analysis, significantly higher HRs for PD were found in the VI groups (low vision, HR: 1.18, 95% CI: 1.12–1.24; blindness, HR: 1.52, 95% CI: 1.28–1.8; *p* < 0.0001; Table 3). Blindness revealed the highest risk for PD, followed by low vision and normal vision. Figure 2 shows the seven-year cumulative incidence of PD, as demonstrated by the development of PD among the participants with normal vision, low vision, and blindness. The 7-year cumulative incidence of PD was highest in participants with blindness (48.17%), followed by those with low vision (36.60%) and normal vision (30.61%; *p* < 0.0001, two-tailed log-rank test).

The HRs for PD as demonstrated by abnormal BSRS-5 scores from the univariate Cox regression analysis model are shown in Table 2. We found that female sex; being single; educational level below senior high school; BMI < 18.5 kg/m^2^; and abnormal cholesterol, TG, and albumin levels were associated with significantly higher HRs in PD. By contrast, male sex, age > 75 years, drinking, BMI of 24–27 kg/m^2^, hypertension, DM, and abnormal globulin levels could be associated with lower HRs for PD.

After adjusting for covariates through multivariate Cox regression analysis (Table 3), participants who were single (divorce/widowed, HR: 1.18, 95% CI: 1.11–1.25; unmarried/separated HR: 1.39, 95% CI: 1.28–1.5), had a lower educational level (elementary/junior high school, HR: 1.19, 95% CI: 1.13–1.26; illiterate HR: 1.46, CI: 1.34–1.58), smoked (HR: 1.25, 95% CI: 1.14–1.38), had a BMI of <18.5 kg/m^2^ (HR: 1.31, 95% CI: 1.19–1.44), and had abnormal blood TG (HR: 1.13, 95% CI: 1.07–1.2) and albumin (HR: 1.24, 95% CI: 1.11–1.37) levels showed significantly higher HRs for PD than the others. By contrast, participants who were older (75–84 years, HR: 0.55, 95% CI: 0.53–0.58; >85 years, HR: 0.45, 95% CI: 0.41–0.49), were male (HR:0.66, 95% CI: 0.63–0.69), had a BMI of >24 kg/m^2^ (BMI = 24–27 kg/m^2^, HR:0.85, 95% CI: 0.81–0.9; BMI > 27 kg/m^2^, HR: 0.9, 95% CI: 0.85–0.96), had hypertension (HR: 0.85, 95% CI: 0.81–0.89) or DM (HR: 0.9, 95% CI: 0.86–0.94), and had abnormal blood cholesterol (HR: 0.94, 95% CI: 0.9–0.98) and globulin (HR: 0.87, 95% CI: 0.83–0.91) levels showed significantly lower HRs for PD than the others.

**Figure 3:**
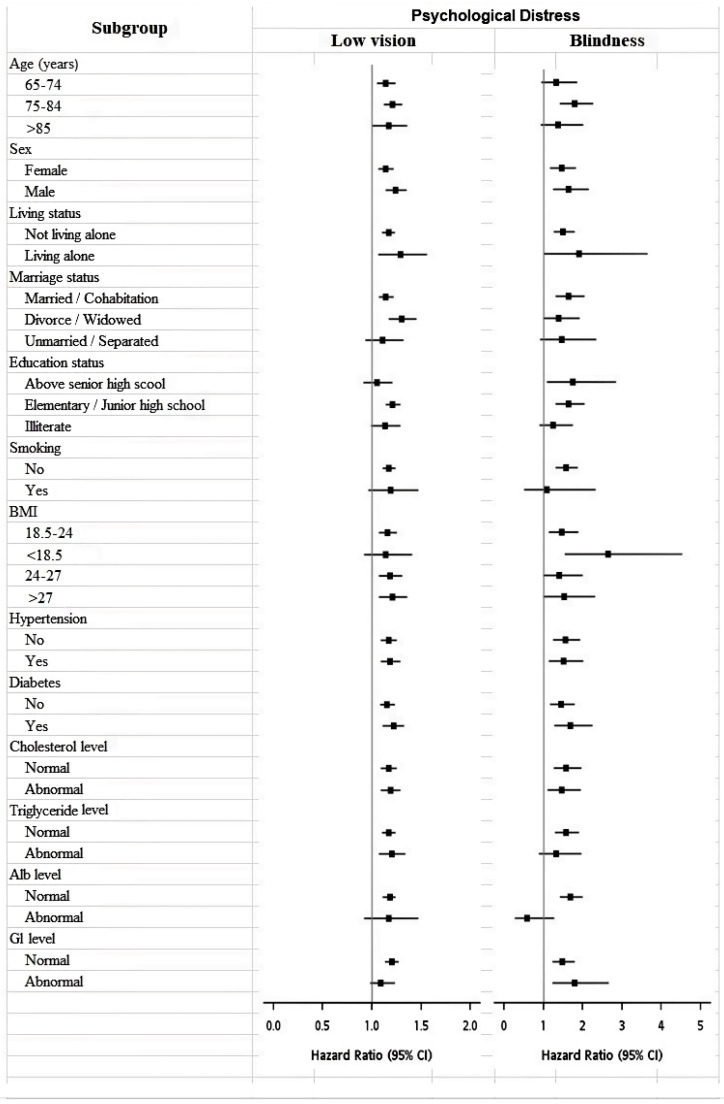
Forest plots for the subgroup analysis of VI in PD as demonstrated by abnormal BSRS-5 scores. HRs are represented by squares, and horizontal lines crossing these squares represent 95% CIs. The HRs for PD were significantly higher in groups with low vision and blindness than in the group without VI. VI = visual impairment; PD = psychological distress; BSRS = Brief Symptom Rating Scale; HR = hazard ratio; CI = confidence interval; BMI = body mass index; Alb = albumin; Gl = globulin.

Figure 3 illustrates the forest plots for the subgroup analysis of VI in PD as demonstrated by abnormal BSRS-5 scores. After stratifying for all other variables, the HRs showed similar trends for each subgroup—the HRs of PD for participants with low vision or blindness were higher than those of participants with normal vision. Participants who were aged 75–84 years (low vision, HR: 1.21, 95% CI: 1.12–1.31; blindness, HR: 1.79, 95% CI: 1.41–2.27), completed elementary or junior high school education only (low vision, HR: 1.21, 95% CI: 1.14–1.29; blindness, HR: 1.64, 95% CI: 1.31–2.05), had a BMI of 18.5–24 kg/m^2^ (low vision, HR: 1.16, 95% CI: 1.07–1.25; blindness, HR: 1.47, 95% CI: 1.14–1.89), and had no smoking habits (low vision, HR: 1.17, 95% CI: 1.11–1.24; blindness, HR: 1.57, 95% CI: 1.31–1.87) showed significantly high HRs for PD in VI.

## 4. Discussion

The primary finding of this study indicates that VI is independently and significantly associated with a higher incidence of PD among older Asian people. Thus, treating pre-existing VI aggressively may prevent PD in the future. The exact mechanisms of this effect may be viewed from several aspects. Ong et al. reported that VI leads to activity restriction, which could cause PD [23,24]. Other previous studies revealed that low wealth and social status increase the risk of developing VI and PD [24,25,26]. In our study, low economic and social support cumulatively increased the risk of VI and accelerated the development of PD. In the normal process of aging, cognitive function may deteriorate [27]. Zheng et al. stated that VI leads to cognitive decline cross-sectionally and longitudinally [28]. Cognitive decline may be a risk factor for PD [29]. Thus, we suggest that VI increases the risk of PD indirectly via cognitive decline. The mechanisms through which VI leads to PD may be directly or indirectly associated with activity restriction, low economic/social support, and cognitive decline. Correcting these factors through medical or social means may help control PD and improve the patient’s overall QOL.

We further evaluated other risk factors for PD in older adults, one of which was age. Patients aged >75 years had a lower risk for PD than those aged <75 years. In addition, patients aged 75–80 years with VI had a significantly higher risk of PD than their counterparts with normal vision. Previous studies reported inconsistent findings regarding the effect of age on PD in older adults. Some studies showed a trend similar to that noted in our study: the prevalence of PD is generally lower in older patients than in younger patients [30,31,32]. However, other studies showed an opposite trend [33,34]. The factors causing age-related differences in PD development are complicated, and they may arise from physical disorders, disability, bereavement, loneliness, institutionalization, and subtle organic or neurochemical brain changes [35]. Therefore, explaining the results based on one or two factors may be difficult. However, we eliminated the potential confounders, including socioeconomic status and baseline health conditions. Further studies with a more comprehensive evaluation are warranted to confirm our findings.

Female sex has long been noted to be a risk factor for PD. Epidemiological studies support our findings and reveal that PD is two to three times more common in women than in men, possibly because women usually base their self-esteem on intimate dyadic relationships, whereas men have broader investments across intimate and social relationships [36]. The effect of sex on PD is not only a physical issue but also involves the social division of labor. Evaluating the effect of different sexes on social conditions may be worth pursuing in future studies.

The risks presented by social status were also noted in our study. The literature indicates that being single, widowed, or separated is directly associated with PD, which was confirmed in our study [37,38,39]. Married partners tend to have better mental health than unmarried individuals on the basis of the support received from the marital relationship [40,41]. Schlax et al. revealed that low educational level and income may be associated with depressive symptoms [42]. Our study further showed that VI patients with elementary or junior high school educations had a significantly higher risk for PD than those with normal vision. Therefore, social support appears to be an important factor that influences PD development.

We further identified smoking as an independent factor associated with PD. Smokers and nonsmokers both showed similar trends for a higher risk of PD in participants with low vision or blindness than in those with normal vision. Fluharty et al. reported that smoking is associated with subsequent depression and anxiety and has direct effects on neurocircuitry that causes patients to be more susceptible to environmental stressors [43]. Therefore, smoking cession may be considered a target to decrease a patient’s risk for PD. In our study, a BMI of <18.5 kg/m^2^ was associated with higher HRs in PD, whereas a BMI of >24 kg/m^2^ was associated with lower HRs. The effect of low BMI on PD was illustrated in our study. A possible explanation for this finding is that low BMI is usually associated with poor nutrition or food intake, which could cause various mental or metabolic problems, eventually leading to PD [44]. Why participants with a high BMI appear to have a lower risk for developing PD remains unknown, but we hypothesize that these individuals may receive better care; thus, they would not immediately manifest the signs of PD. More studies on the effects of lifestyle on PD are needed to confirm our hypothesis.

Regarding general health conditions, hypertension, DM, and abnormal cholesterol were associated with lower HRs in PD. This finding is interesting because these metabolic syndromes can lead to fatal diseases such as coronary artery disease or ischemic stroke. Bădescu et al. advocated that stress and inflammatory processes induced by these metabolic symptoms may cause PD [45]. Chan et al. supported this viewpoint [46]. We hypothesize that the participants in our study might not have suffered from severe complications of these diseases because they voluntarily submitted to the annual health examination. Thus, other detailed risk factors for PD must be clarified in future studies.

Our study has several limitations. First, although the BSRS-5 scores could efficiently reveal mental health problems during a general health examination, the questionnaire might have been biased, particularly in older adults. Second, although mental health problems were detected in our study, the determination of their exact etiology is technically challenging. Thus, categorizing different mental diseases was difficult. Different types of PD, for example, depression or anxiety, may have different etiologies or pathways. The further investigation of causal relationships may yield more precise results. In summary, this study clearly demonstrates the effect of VI on the psychological status of patients. We suggest that early referral for VI examination and treatment should be implemented in clinical practice, particularly among patients suffering from psychological problems.

## 5. Conclusions

VI is independently and significantly associated with a higher incidence of PD among older Asian people. In addition, being single, having a low educational level, being a smoker, having a BMI of <18.5 kg/m^2^, and abnormal systemic personal health conditions are all associated with PD. Therefore, identifying and correcting the modifiable risk factors of PD are important measures to improve the overall QOL of older adults and to relieve social burdens.

## Figures and Tables

**Table 1:** Demographic information of the elderly participants with normal and abnormal Brief Symptom Rating Scale-5 scores during physical examination in 2005–2011. BMI = body mass index; Alb = albumin; Gl = globulin.

Factors	Normal (*n* = 94,384)	Abnormal (*n* = 10,708)	*p*-Value
Sight			<0.0001
Normal sight	74,818 (79.27)	8067 (75.34)	
Low vision	18,421 (19.52)	2454 (22.92)	
Blindness	1145 (1.21)	187 (1.75)	
Age (years)			<0.0001
65–74	46,611 (49.43)	5367 (50.16)	
75–84	37,713 (39.99)	4360 (40.75)	
>85	9973 (10.58)	973 (9.09)	
Sex			<0.0001
Male	45,230 (47.92)	6586 (61.51)	
Female	49,154 (52.08)	4122 (38.49)	
Living status			<0.0001
Not living alone	89,312 (94.63)	10,028 (93.65)	
Living alone	5066 (5.37)	680 (6.35)	
Marriage status			<0.0001
Married/Cohabitation	69,124 (74.34)	7397 (69.94)	
Divorce/Widowed	15,502 (16.67)	2185 (20.66)	
Unmarried/Separated	8359 (8.99)	994 (9.40)	
Education status			<0.0001
Illiterate	23,421 (28.44)	1953 (21.17)	
Elementary/Junior high school	51,975 (63.11)	6154 (66.70)	
Above senior high school	6962 (8.45)	1120 (12.14)	
Smoking			0.026
No	88,169 (93.62)	10,065 (94.17)	
Yes	6012 (6.38)	623 (5.83)	
Drinking			0.013
No	92,115 (97.83)	10,492 (98.19)	
Yes	2047 (2.17)	193 (1.81)	
Betel nut consumption			0.293
No	93,612 (99.56)	10,623 (99.63)	
Yes	410 (0.44)	39 (0.37)	
BMI			<0.0001
<18.5	3801 (4.05)	609 (5.73)	
18.5–24	42,191 (44.93)	4999 (47.07)	
24–27	29,607 (31.53)	2995 (28.20)	
>27	18,302 (19.49)	2018 (19.00)	
Hypertension			<0.0001
No	58,034 (61.58)	6973 (65.27)	
Yes	36,202 (38.42)	3710 (34.73)	
Diabetes			<0.0001
No	57,929 (61.53)	6953 (65.07)	
Yes	36,221 (38.47)	3733 (34.93)	
Cholesterol level			0.451
Normal	55,297 (58.78)	6234 (58.40)	
Abnormal	38,782 (41.22)	4441 (41.60)	
Triglyceride level			<0.0001
Normal	76,262 (81.06)	8474 (79.34)	
Abnormal	17,823 (18.94)	2206 (20.66)	
Alb level			<0.0001
Normal	84,826 (95.38)	9476 (94.50)	
Abnormal	4112 (4.62)	552 (5.50)	
Gl level			<0.0001
Normal	67,739 (76.04)	7915 (78.67)	
Abnormal	21,340 (23.96)	2146 (21.33)	

**Table 2:** Univariate Cox regression analysis of factors associated with psychological distress among the elderly with normal vision, low vision, and blindness. CI = confidence interval; BMI = body mass index; Alb = albumin; Gl = globulin.

Factors	Univariate	
	Hazard Ratio (95% CI)	*p*-Value
Sight		
Normal	1	
Low vision	1.16 (1.11–1.22)	<0.0001
Blindness	1.59 (1.37–1.83)	<0.0001
Age (years)		
65–74	1	
75–84	0.58 (0.55–0.6)	<0.0001
>85	0.49 (0.46–0.53)	<0.0001
Sex		
Female	1	
Male	0.56 (0.54–0.58)	<0.0001
Living status		
Not living alone	1	
Living alone	1.01 (0.94–1.09)	0.754
Marriage status		
Married/Cohabitation	1	
Divorce/Widowed	1.29 (1.23–1.35)	<0.0001
Unmarried/Separated	1.31 (1.23–1.4)	<0.0001
Education status		
Above senior high scool	1	
Elementary/Junior high school	1.44 (1.37–1.51)	<0.0001
Illiterate	1.93 (1.8–2.08)	<0.0001
Smoking		
No	1	
Yes	1.08 (0.99–1.17)	0.062
Drinking		
No	1	
Yes	0.83 (0.72–0.95)	0.008
Betel nut consumption		
No	1	
Yes	1.16 (0.85–1.59)	0.351
BMI		
18.5–24	1	
<18.5	1.32 (1.21–1.43)	<0.0001
24–27	0.87 (0.83–0.91)	<0.0001
>27	0.95 (0.91–1.01)	0.069
Hypertension		
No	1	
Yes	0.82 (0.79–0.85)	<0.0001
Diabetes		
No	1	
Yes	0.87 (0.83–0.9)	<0.0001
Cholesterol level		
Normal	1	
Abnormal	1.08 (1.04–1.12)	<0.0001
Triglyceride level		
Normal	1	
Abnormal	1.14 (1.08–1.19)	<0.0001
Alb level		
Normal	1	
Abnormal	1.22 (1.12–1.33)	<0.0001
Gl level		
Normal	1	
Abnormal	0.88 (0.84–0.93)	<0.0001

**Table 3:** Multivariate Cox regression analysis of factors associated with psychological distress among the elderly with normal vision, low vision, and blindness. CI = confidence interval; BMI = body mass index; Alb = albumin; Gl = globulin.

Factors	Multivariate	
	Hazard Ratio (95% CI)	*p*-Value
Sight		
Normal	1	
Low vision	1.18 (1.12–1.24)	<0.0001
Blindness	1.52 (1.28–1.8)	<0.0001
Age (years)		
65–74	1	
75–84	0.55 (0.53–0.58)	<0.0001
>85	0.45 (0.41–0.49)	<0.0001
Sex		
Female	1	
Male	0.66 (0.63–0.69)	<0.0001
Living status		
Not living alone	1	
Living alone	0.95 (0.87–1.04)	0.294
Marriage status		
Married/Cohabitation	1	
Divorce/Widowed	1.18 (1.11–1.25)	<0.0001
Unmarried/Separated	1.39 (1.28–1.5)	<0.0001
Education status		
Above senior high school	1	
Elementary/Junior high school	1.19 (1.13–1.26)	<0.0001
Illiterate	1.46 (1.34–1.58)	<0.0001
Smoking		
No	1	
Yes	1.25 (1.14–1.38)	<0.0001
Drinking		
No	1	
Yes	0.96 (0.81–1.14)	0.636
Betel nut consumption		
No	1	
Yes	0.78 (1.05–0.74)	0.784
BMI		
18.5–24	1	
<18.5	1.31 (1.19–1.44)	<0.0001
24–27	0.85 (0.81–0.9)	<0.0001
>27	0.9 (0.85–0.96)	0.0007
Hypertension		
No	1	
Yes	0.85 (0.81–0.89)	<0.0001
Diabetes		
No	1	
Yes	0.9 (0.86–0.94)	<0.0001
Cholesterol level		
Normal	1	
Abnormal	0.94 (0.9–0.98)	0.005
Triglyceride level		
Normal	1	
Abnormal	1.13 (1.07–1.2)	<0.0001
Alb level		
Normal	1	
Abnormal	1.24 (1.11–1.37)	<0.0001
Gl level		
Normal	1	
Abnormal	0.87 (0.83–0.91)	<0.0001

## Data Availability

The data that support the findings of this study are available from the corresponding author upon reasonable request.
